# Supplementary figures and images for: A 24-year longitudinal study on a STEM gateway general chemistry course and the reduction of achievement disparities
Source: PLoS One. 2025 Feb 26;20(2):e0318882. doi: 10.1371/journal.pone.0318882 (PMC11864549; doi:10.1371/journal.pone.0318882)

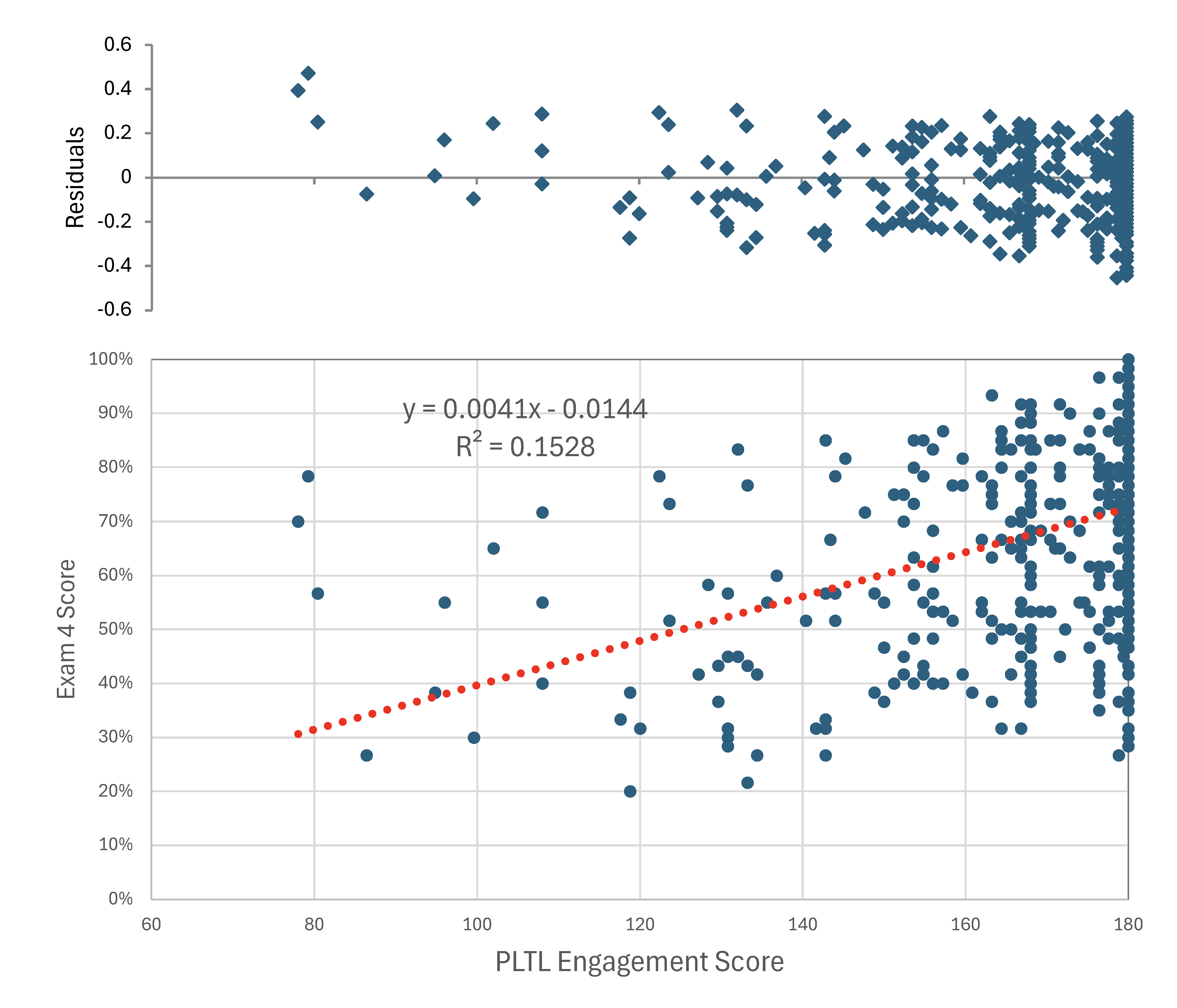

Supplement: S1 Fig — The residuals plot shows that heteroscedasticity is not apparent and implies the fitting satisfies validity criteria. The equation for the linear regression line (red) is shown. In the PLTL sections, leaders assess students’ engagement with content preparation, problem-solving, participation, and post-section worksheets. (TIFF) [file pone.0318882.s001.tiff]

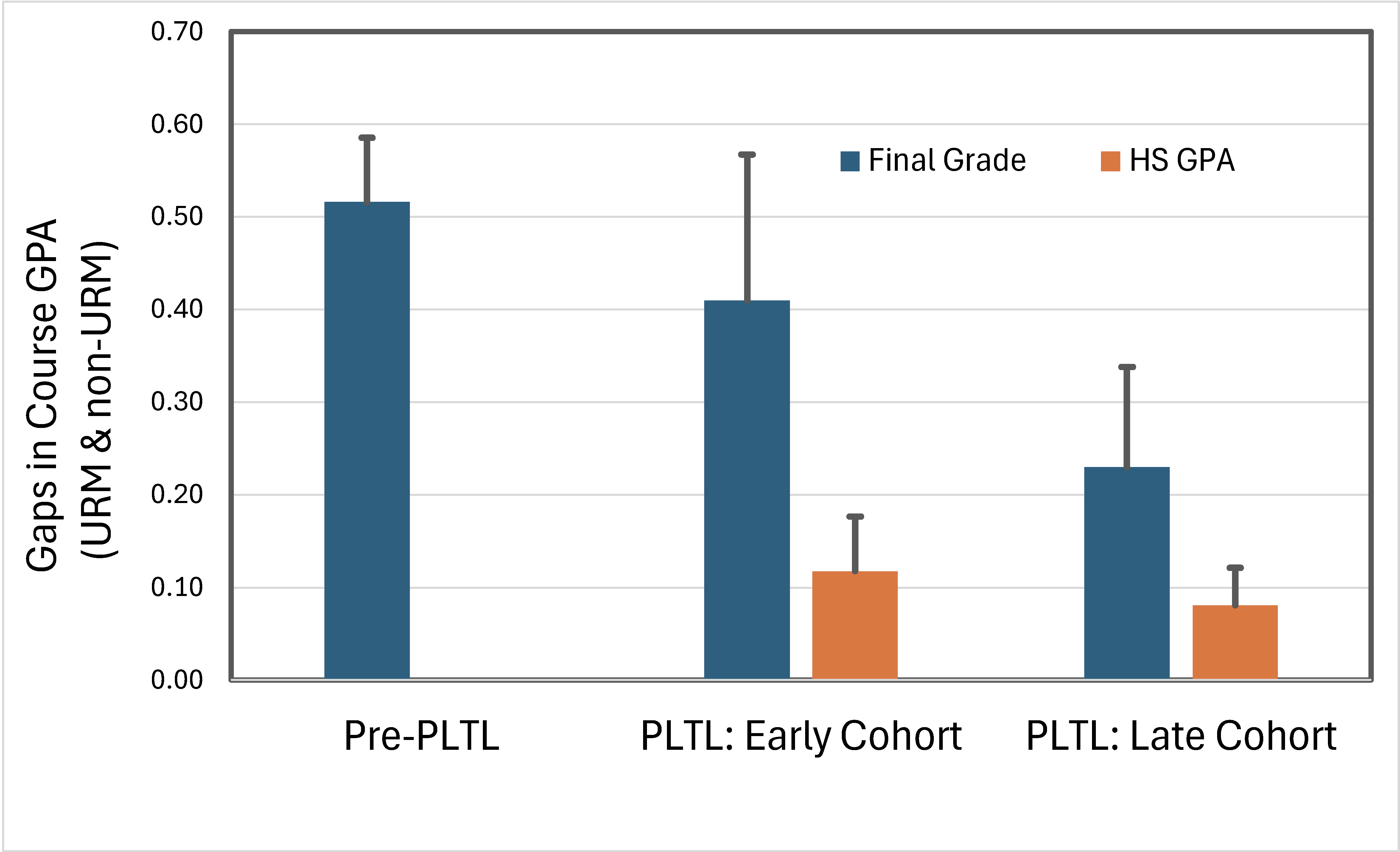

Supplement: S2 Fig — The HS GPA shown in orange bars indicates the gaps in those averages in the two cohort periods. There is insufficient data for pre-PLTL comparisons with HS-GPA. (TIFF) [file pone.0318882.s002.tiff]

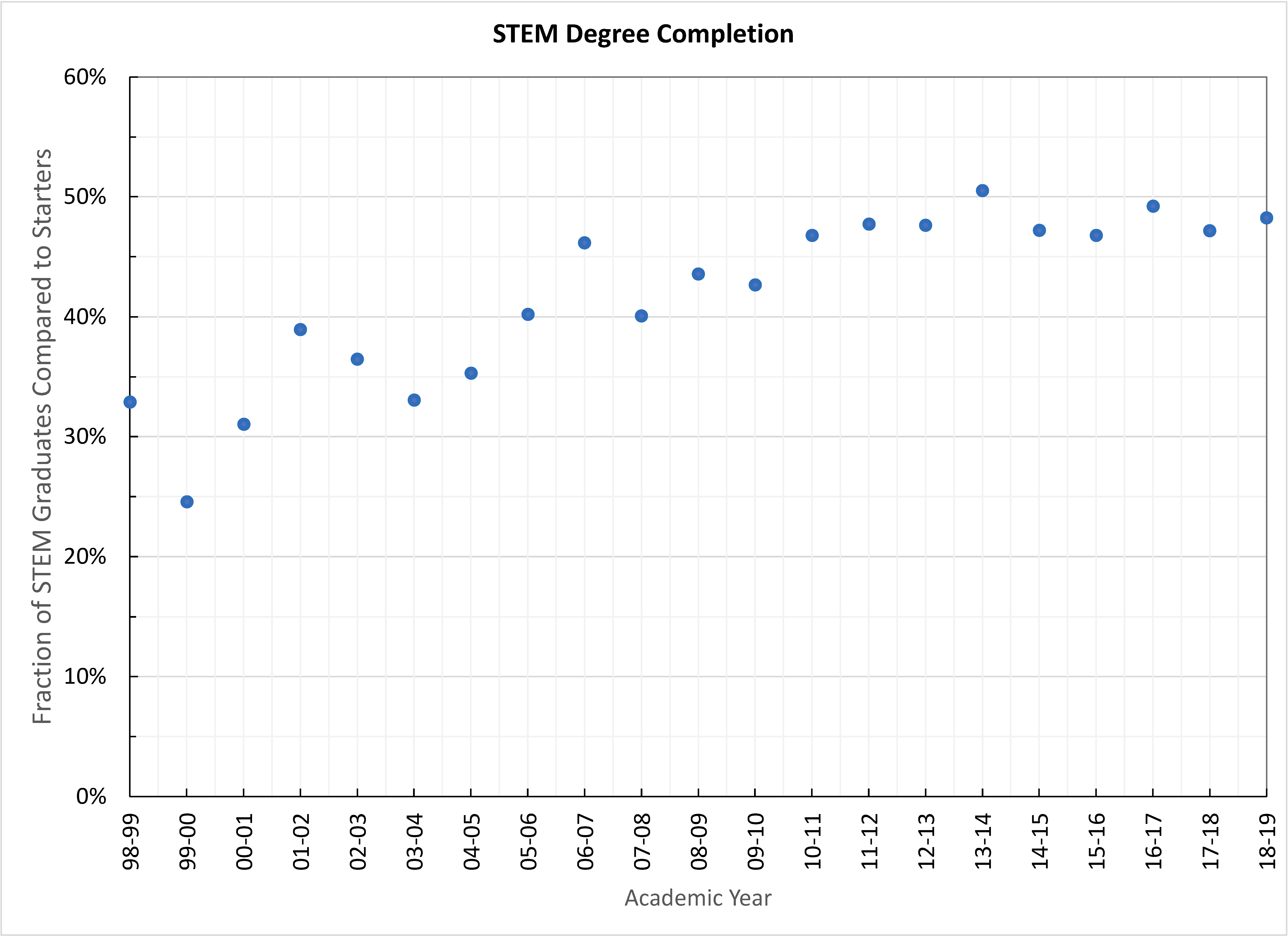

Supplement: S3 Fig — The figure shows the fraction of students with initial STEM interest that successfully complete a STEM degree program (in a six-year window). The PLTL program started in Fall, 1998, so the first impact of PLTL would likely be starting in AY 2002–03 and later. (TIFF) [file pone.0318882.s003.tiff]
