# Supplementary material for: A 24-year longitudinal study on a STEM gateway general chemistry course and the reduction of achievement disparities
Source: PLoS One. 2025 Feb 26;20(2):e0318882. doi: 10.1371/journal.pone.0318882 (PMC11864549; doi:10.1371/journal.pone.0318882)
Supplement: S1 Table — (DOCX) [file pone.0318882.s004.docx]

***S1.Table. Course-level statistics (1996-2019).***

| ***Variable*** | ***N (sections)*** | ***Percentage/Mean*** |
| --- | --- | --- |
| PLTL | 78 | 91.7% |
| No PLTL | 7 | 8.3% |
| **Term** | | |
| Spring | 27 | 31.8% |
| Fall | 58 | 68.2% |
| **Placement Tests** | | |
| ACS Toledo Placement Exam | 51 | 60.0% |
| ALEKS Math only | 14 | 16.5% |
| ALEKS Math and Chemistry PE**^*^** | 20 | 20.5% |
| **Additional** | | |
| Average SAT score | 85 | 1154 |
| Percent Female | 85 | 49.5% |
| Percent First Generation | 85 | 30.6% |
| Percent URM^†^ | 85 | 18.8% |
| *Internal IUPUI Chemistry Department Placement Examination.  †Underrepresented includes African American, Hispanic/Latine, Native American, Native Hawaiian/Pacific Islander, or two or more races. | | |
