# Supplementary material for: A 24-year longitudinal study on a STEM gateway general chemistry course and the reduction of achievement disparities
Source: PLoS One. 2025 Feb 26;20(2):e0318882. doi: 10.1371/journal.pone.0318882 (PMC11864549; doi:10.1371/journal.pone.0318882)
Supplement: S2 Table — (DOCX) [file pone.0318882.s005.docx]

***S1.Table. Course-level statistics (1996-2019).***

| ***Variable*** | ***N (sections)*** | ***Percentage/Mean*** |
| --- | --- | --- |
| PLTL | 78 | 91.7% |
| No PLTL | 7 | 8.3% |
| **Term** | | |
| Spring | 27 | 31.8% |
| Fall | 58 | 68.2% |
| **Placement Tests** | | |
| ACS Toledo Placement Exam | 51 | 60.0% |
| ALEKS Math only | 14 | 16.5% |
| ALEKS Math and Chemistry PE**^*^** | 20 | 20.5% |
| **Additional** | | |
| Average SAT score | 85 | 1154 |
| Percent Female | 85 | 49.5% |
| Percent First Generation | 85 | 30.6% |
| Percent URM^†^ | 85 | 18.8% |
| *Internal IUPUI Chemistry Department Placement Examination.  †Underrepresented includes African American, Hispanic/Latine, Native American, Native Hawaiian/Pacific Islander, or two or more races. | | |

***S2. Table. Effect of PLTL on course-level DFW grades.***

| ***Variable*** | ***SS III*** | ***DF*** | ***MS*** | ***F*** | ***η^2‡^*** | ***ω^2‡^*** |
| --- | --- | --- | --- | --- | --- | --- |
| PLTL term | 0.063 | 1 | 0.063 | 11.93† | 0.137 | 0.114 |
| Spring Term | 0.003 | 1 | 0.003 | 0.65 | 0.009 | -0.004 |
| URM^*^ | 0.020 | 1 | 0.020 | 3.72 | 0.047 | 0.031 |
| Average SAT | 0.131 | 1 | 0.131 | 24.96† | 0.250 | 0.220 |
| Female | 0.016 | 1 | 0.016 | 3.11 | 0.040 | 0.024 |
| First Gen | 0.003 | 1 | 0.003 | 0.65 | 0.009 | -0.004 |
| Pell | < 0.001 | 1 | < 0.001 | 0.03 | < 0.001 | -0.011 |
| Placement Tests | 0.016 | 2 | 0.008 | 1.55 | 0.040 | 0.013 |
| Error | 0.395 | 75 | 0.005 |  |  |  |
| ^*^ URM: Black, Hispanic/Latine, Native American, Native Hawaiian/Pacific Islander, or Two or more races, R^2^ = 0.65;  † Statistically significant at 𝛼 < 0.01;  ‡ Small effect for partial eta squared, *η*^2^ = 0.01; medium effect = 0.06; large effect = 0.14. Similarly, effect sizes for *ω***^2^.** See reference Cohen. (*2*) | | | | | | |
