# Supplementary material for: A 24-year longitudinal study on a STEM gateway general chemistry course and the reduction of achievement disparities
Source: PLoS One. 2025 Feb 26;20(2):e0318882. doi: 10.1371/journal.pone.0318882 (PMC11864549; doi:10.1371/journal.pone.0318882)
Supplement: S3 Table — (DOCX) [file pone.0318882.s006.docx]

***S3. Table. Marginal means for PLTL and non-PLTL course sections***

|  | ***Mean DFW grades*** | ***Marginal means**** |
| --- | --- | --- |
| Pre-PLTL | 51.8% | 47.2% |
| Include PLTL | 30.1% | 31.3% |
| All | 31.6% |  |
| * Marginal means adjusted for the effects of term, average SAT score, % female, % first gen, % URM, % Pell, and placement test administered. | | |
