# Supplementary material for: A 24-year longitudinal study on a STEM gateway general chemistry course and the reduction of achievement disparities
Source: PLoS One. 2025 Feb 26;20(2):e0318882. doi: 10.1371/journal.pone.0318882 (PMC11864549; doi:10.1371/journal.pone.0318882)
Supplement: S4 Table — (DOCX) [file pone.0318882.s007.docx]

**S4.** ***Table. Student and course level statistics for variables included in the random intercept model.***

| ***Variable*** | ***N*** | ***Mean (Std. Dev.)*** |
| --- | --- | --- |
|  | **Student level** | |
| Ex_3+4_ | 3,873 | 161.6 (39.0) |
| PLTL* | 3,873 | 168.5 (21.1) |
| Incoming HS/Transfer GPA | 3,873 | 3.44 (1.16) |
| URM | 3,873 | 18.6% |
| Pell | 3,873 | 29.9% |
|  | **Course level** | |
| Spring term sections | 29 | 27.6% |
| Recent sections AY 2016-2018 and Fall 2019 | 29 | 65.5% |
| * Some sections required a small renormalization that was less than 3% which would not negatively impact the overall results. | | |
