# Supplementary material for: A 24-year longitudinal study on a STEM gateway general chemistry course and the reduction of achievement disparities
Source: PLoS One. 2025 Feb 26;20(2):e0318882. doi: 10.1371/journal.pone.0318882 (PMC11864549; doi:10.1371/journal.pone.0318882)
Supplement: S5 Table — (DOCX) [file pone.0318882.s008.docx]

**S5. Table. *Random intercept model: Effect of PLTL engagement on the Ex_3+4_ score****

| ***Parameter*** | ***Null model*** | ***Final model (Standard error)*** |
| --- | --- | --- |
|  | **Fixed effect (β, standard error)** | |
| Intercept | 158.61 (3.01) | 149.23 (2.65) |
| PLTL^†^ |  | 0.56^‡^ (0.04) |
| Incoming HS/Transfer GPA^∥^ |  | 15.36^‡^ (1.53) |
| SAT^¶^ |  | 0.82^‡^ (0.06) |
| Section if Spring term |  | -2.45 (2.93) |
| Recent Sections, AY 2016-2018 and Fall 2019 |  | 13.81^‡^ (3.03) |
|  | **Random effects** | |
| Level 2 Between Class effect | 240.54 (71.75) | 52.71 (19.28) |
| Level 1 Residual | 1,366 (31.96) | 932.85 (22.36) |
| Intraclass correlation | 0.150 | 0.112 |
| **Random Intercept Model:* Level 1: Y_ij_ = β_0j_ [intercept] + 0.56 × (centered PLTL engagement score) + 15.36 × (centered  incoming GPA) + 0.82 × (centered SAT) + ε_ij_, and Level 2: β_0j_ = 149.23 – 2.45 (Spring offering) + 13.81 (recent section).  † Centered on PLTL average (168.5).  ‡ *p* < 0.01  ∥ Centered on average high school and transfer GPA (3.44);  ¶ Centered on average SAT score (1189)  R^2^: Level 1, 0.3005 and Level 2, 0.7496. Chi-square Level 1 =  932.85 (significant at *p* < 0.01); *n* = 3,578 | | |
