# Supplementary material for: A 24-year longitudinal study on a STEM gateway general chemistry course and the reduction of achievement disparities
Source: PLoS One. 2025 Feb 26;20(2):e0318882. doi: 10.1371/journal.pone.0318882 (PMC11864549; doi:10.1371/journal.pone.0318882)
Supplement: S6 Table — (DOCX) [file pone.0318882.s009.docx]

**S6.** **Table. *OLS Regression on Ex_3+4_ score for URM students only.****

| ***Parameter*** | **β *Coefficients (Standard error)*** |
| --- | --- |
| Intercept | 133.44 (3.93) |
| PLTL† | 0.61* (0.06) |
| Incoming GPA^‡^ | 3.34* (0.85) |
| Received Pell Grant | -6.85* (2.49) |
| Section in Spring term | -5.62 (2.53) |
| Sections, AY 2016-2018 and Fall 2019 | 24.91* (3.74) |
| OLS: Regression including only URM Students (Black, Hispanic/Latine, Native American, Native Hawaiian/Pacific Islander, or two or more races) including coefficients. Y [Ex_3+4_] = 133.44 + 0.61 x_1_[PLTL engagement score] + 3.43x_2_ [incoming GPA] – 6.85x_3_ [Receipt of a Pell grant] – 5.62x_4_ [course term] + 24.91x_5_ [course was recent] + ε  * *p* < 0.01  † Centered on PLTL average (168.5)  ‡ Centered on average high school/ GPA (3.44); R^2^ = transfer 0.220; *n* = 721 | |
