# Supplementary material for: A 24-year longitudinal study on a STEM gateway general chemistry course and the reduction of achievement disparities
Source: PLoS One. 2025 Feb 26;20(2):e0318882. doi: 10.1371/journal.pone.0318882 (PMC11864549; doi:10.1371/journal.pone.0318882)
Supplement: S7 Table — (DOCX) [file pone.0318882.s010.docx]

***S7. Table. The OLS Regression on the Ex_3+4_ score for Pell recipients.****

| ***Parameter*** | **β *Coefficients (Standard error)*** |
| --- | --- |
| Intercept | 144.36 (2.70) |
| PLTL^‡^ | 0.57† (0.05) |
| Incoming GPA^§^ | 1.90† (0.73) |
| URM^∥^ | -8.15† (2.20) |
| Section in Spring term | -2.06 (2.14) |
| Sections, AY 2016-2018 and Fall 2019 | 19.70† (2.87) |
| ** OLS Regression including only Pell recipients:* Y [Ex_3+4_ score] = 144.36 + 0.57 x_1_[PLTL engagement score] + 1.90x_2_ [incoming GPA] – 8.15x_3_ [URM] - 2.06x_4_ [Spring term] + 19.70x_5_ [course was recent] + ε;  † *p* < 0.01;  ‡ Centered on PLTL average (168.5);  § Centered on average high school and transfer GPA (3.44);  ∥ URM = Black, Hispanic, Native American, Native Hawaiian/Pacific Islander, or Two or more races; R^2^ = 0.166; *n* = 1158. Non-URM are white or Asian (or undeclared). | |
