# Supplementary material for: A 24-year longitudinal study on a STEM gateway general chemistry course and the reduction of achievement disparities
Source: PLoS One. 2025 Feb 26;20(2):e0318882. doi: 10.1371/journal.pone.0318882 (PMC11864549; doi:10.1371/journal.pone.0318882)
Supplement: S8 Table — (DOCX) [file pone.0318882.s011.docx]

***S8. Table. Comparison Metrics (Pre-Enrollment, Internal, and Output) for Early and Late Cohorts.***

| **Selected Performance Metrics** | **Fall Early Cohort  (2002-05)** | **Fall Late Cohort  (2016-19)** | **% Change^†^** | **∆^‡^** |
| --- | --- | --- | --- | --- |
|  | *n* =1266 | *n* = 2079 |  |  |
| **Pre-Enrollment Student Preparation** | | | |  |
| Course SAT, averaged cohort* (percentile) | 1164 (73^rd^) | 1219 (77^th^) | +5% | 55 |
| Course HS GPA, averaged cohort* | 3.339 | 3.650 | +10% | +0.31 |
| **Internal: Course Metrics** | | | |  |
| Ex_3+4_ scores, averaged cohort* | 61% | 71% | +16% | +10% |
| Final Exam scores, averaged cohort* | 55% | 64% | +16% | +9% |
| **Output: Course Metrics** | | | |  |
| Course GPA, averaged cohort* | 2.49 | 2.72 | +8% | +.23 |
| Course total points at median | 67.8% | 78.6% | 16% | 11% |
| Median Course Grade (50^th^ percentile) | 2.3 | 2.7 |  |  |
| %ABC, averaged cohort* | 79% | 84% | +7% | +5% |
| %DFW, averaged cohort* | 37% | 22% | –39% | –15% |

* Combined cohort average differences, *p* < 0.05

^†^ Fractional change from Early to Late cohorts, (X_Late_ – X_Early_)/X_Early_.

^‡^ Numerical difference between Early and Late cohort averages.
