# Supplementary material for: A 24-year longitudinal study on a STEM gateway general chemistry course and the reduction of achievement disparities
Source: PLoS One. 2025 Feb 26;20(2):e0318882. doi: 10.1371/journal.pone.0318882 (PMC11864549; doi:10.1371/journal.pone.0318882)
Supplement: S9 Table — (DOCX) [file pone.0318882.s012.docx]

**S9.** ***Table. Detailed Comparative Performance within Cohorts.***

| **Fall Early Cohort** | | | | | **Fall Late Cohort** | | | | |
| --- | --- | --- | --- | --- | --- | --- | --- | --- | --- |
| AY* | *n†* | Avg  HS GPA | Average Final Exam Score | Average Course GPA (median^‡^) | AY* | *n†* | Average HS GPA | Average Final Exam Score | Average Course GPA (median^‡^) |
| 2002 | 293 | 3.265 | 51.9% | 2.51 (2.3) | 2016 | 623 | 3.616 | 61.9% | 2.74 (3.0) |
| 2003 | 379 | 3.266 | 53.9% | 2.42 (2.3) | 2017 | 642 | 3.615 | 61.3% | 2.66 (3.0) |
| 2004 | 315 | 3.374 | 56.4% | 2.48 (2.3) | 2018 | 663 | 3.694 | 63.6% | 2.76 (3.0) |
| 2005 | 281 | 3.410 | 58.4% | 2.55 (2.3) | 2019 | 599 | 3.669 | 67.7% | 2.74 (3.0) |

* AY = Academic Year

† *n* applies to “Average Final Exam Score” and “Average Course GPA” data only.

‡ The *median* is the 50^th^ percentile grade in the section.
