# Supplementary material for: A 24-year longitudinal study on a STEM gateway general chemistry course and the reduction of achievement disparities
Source: PLoS One. 2025 Feb 26;20(2):e0318882. doi: 10.1371/journal.pone.0318882 (PMC11864549; doi:10.1371/journal.pone.0318882)
Supplement: S10 Table — (DOCX) [file pone.0318882.s013.docx]

***S10.*** ***Table. Comparative Performances (Fall Early vs. Fall Late Cohorts) of URM and non-URM student Final Exam scores, course grades, course DFW rates, and course ABC rates. Fall terms only.***

|  | Early Cohort *n* | Late Cohort *n* | Average Final Exam Score %Change^‡^ | Average Performance Points* %Change^‡^ | Average Course GPA %Change^‡^ | DFW% ∆^†^ | ABC% ∆^†^ | HS GPA %^‡^ |
| --- | --- | --- | --- | --- | --- | --- | --- | --- |
| All | 1268 | 2079 | +16% | +22% | +10% | -15% | +6% | 12% |
| Non-URM | 1120 | 1695 | +16% | +22% | +9% | -15% | +5% | 12% |
| URM | 148 | 384 | +21% | +25% | +19% | -22% | +13% | 13% |

* Student Performance points are Total Course Points – PLTL Engagement score

^†^ Numerical difference in rates.

^‡^ Fractional change in early cohort vs late cohort, (X_Late_ – X_Early_)/X_Early_
