# Supplementary material for: A 24-year longitudinal study on a STEM gateway general chemistry course and the reduction of achievement disparities
Source: PLoS One. 2025 Feb 26;20(2):e0318882. doi: 10.1371/journal.pone.0318882 (PMC11864549; doi:10.1371/journal.pone.0318882)
Supplement: S11 Table — (DOCX) [file pone.0318882.s014.docx]

**S11.** ***Table. Fall Early/Late Cohort Output Metrics: %ABC and %DFW with URM Status.***

| Early Cohort | *n* | %ABC | %DFW |
| --- | --- | --- | --- |
| All | 1728 | 78.8% | 33.0% |
| Non-URM | 1503 | 80.5% | 31.0% |
| URM | 225 | 66.1% | 46.5% |
| Non-URM/URM Gap |  | 14.4% | -15.5% |
| Late Cohort | | | |
| All | 2373 | 84.3% | 17.7% |
| Non-URM | 1917 | 85.5% | 16.3% |
| URM | 456 | 79.3% | 23.9% |
| Non-URM/URM Gap |  | 6.2% | -7.6% |
