# Supplementary material for: A 24-year longitudinal study on a STEM gateway general chemistry course and the reduction of achievement disparities
Source: PLoS One. 2025 Feb 26;20(2):e0318882. doi: 10.1371/journal.pone.0318882 (PMC11864549; doi:10.1371/journal.pone.0318882)
Supplement: Attachment — Submitted filename: Revision_Letter.docx [file pone.0318882.s015.docx]

Luís A. Nunes Amaral, Ph.D.

Academic Editor,

PLOS ONE

January 2, 2025

Dear Dr. Amaral,

Thank you and your staff for handling our manuscript (PONE-D-24-42866). We have revised the manuscript by addressing all editorial comments and those made by the reviewer. The revised manuscript files, both in tracked change and all accepted changes, have been uploaded using the online portal. Below, I outline how the revision has been made.

Reviewer 1.

We appreciate the comments about the quality and rigor of this investigation. The reviewer suggested two revisions, and both have been made. First, we have added more descriptions of the principles of PLTL and included additional references as suggested by the reviewer. This should help curious readers to find more information about PLTL. Second, starting with the abstract, we clarified the meaning of %DFW and %ABC.

Journal requirements.

1. We have formatted the manuscript according to the PLOS guidelines. This includes changing the headings according to the guidelines., placing the figure captions at appropriate locations, and placing the supporting information according to the guidelines.
2. Our study does not include any medical records or patient information; thus, no informed consent is needed.
3. The competing interest section has been revised as suggested.
4. We have the data available to anyone interested in this study in an anonymized format. Unlike the structural or genomic databases, to our knowledge, there is no repository to submit our data. If, however, data can be deposited to the PLOS server, we would welcome that option.
5. We have included the IRB protocol information in the methods section as suggested.
6. We have reviewed the references, and to our knowledge, none has been retracted.

The authors have declared that no competing interests exist.

As suggested, all four figures in the manuscript have been verified by the Preflight Analysis and Conversion Engine (PACE) digital diagnostic tool. We look forward to your decision on this manuscript. Please direct all communications to me, and have a very Happy New Year!

Sincerely,

Partha Basu
